# 3D Cartesian ultrashort double half‐echo imaging of the lung parenchyma for water density imaging

**DOI:** 10.1002/mrm.30615

**Published:** 2025-09-16

**Authors:** Richard B. Thompson, Christopher Keen, Richard Coulden, Hefin Jones, Robert W. Stobbe, Justin G. Grenier

**Affiliations:** ^1^ Department of Radiology and Diagnostic Imaging University of Alberta Edmonton Alberta Canada; ^2^ Department of Biomedical Engineering University of Alberta Edmonton Alberta Canada

**Keywords:** lung, lung water density (LWD), magnetic resonance imaging, non‐Cartesian, pulmonary edema, UTE

## Abstract

**Purpose:**

Develop and illustrate a 3D double half‐echo Cartesian UTE method for spin‐density weighted imaging of the lung parenchyma and calculation of lung water density (LWD).

**Methods:**

A 3D gradient‐echo pulse sequence was modified to acquire half‐echoes, to enable UTEs (TE/TR = 145 μs/1.2 ms), with an acquired resolution of 3.125 mm by 3.125 mm by 5 mm. Breath‐hold (12.9 s) and free‐breathing (94 s) acquisitions, using a center of k‐space navigator, were compared to a previously validated yarnball UTE sequence (1.5T/2.89T). Apparent SNR in the lung parenchyma was measured for all in‐vivo acquisitions. Illustrative clinical cases included heart failure and sarcoidosis with a comparison to CT images.

**Results:**

Lung image quality and calculated LWD was similar for all compared methods at 1.5T and 2.89T for breath‐hold and free‐breathing acquisitions (*N* = 10, *p* > 0.05), with no visible artifacts. The mean lung parenchyma SNR values were 18.4 ± 1.4, 21.8 ± 1.7 and 15.1 ± 1.0 for 1.5T free‐breathing, 2.89T free‐breathing and 2.89T breath‐hold, respectively, and 20.7 ± 1.1 for yarnball acquisitions (2.89T), with corresponding average LWD values of 26.7 ± 2.9%, 27.1 ± 2.5%, 27.1 ± 2.1% and 27.7 ± 2.7%. MRI LWD images and CT scans yielded similar image contrast and normalized signal intensity units. All Cartesian UTE images were reconstructed on the scanner without the requirement for gridding.

**Conclusions:**

A double half‐echo Cartesian UTE pulse sequence provides water‐density weighted images of the lung parenchyma in a breath‐hold or short free‐breathing acquisition with sufficient signal to noise for quantification of LWD at 1.5T or 2.89T.

## 1 INTRODUCTION

Imaging of the lungs is essential for the diagnosis, therapeutic planning and monitoring of respiratory and cardiopulmonary disease. Proton (^1^H) MRI offers three‐dimensional (3D) evaluation of lung structure with similar contrast to CT.^1^ However, quantitative imaging of the lung parenchyma with MRI is challenging due to low proton density and short $T_{2}^{*}$ values of ˜1.5 ms at 1.5T and <1 ms at the 3.0T field strength.^2^, ^3^ Imaging with UTE minimize $T_{2}^{*}$‐related signal loss in the parenchyma and is essential for quantification of lung water density (LWD), for applications such as evaluation of pulmonary edema.^4^, ^5^, ^6^, ^7^, ^8^ Currently, non‐Cartesian acquisitions are required for UTE imaging, with several reported 3D center‐out k‐space trajectories applied in the lungs including radial,^9^ stack of spirals,^10^, ^11^ cones,^12^ and yarnball^13^, ^14^ patterns, for example. These methods require Cartesian gridding of k‐space data for image reconstruction, which is computationally demanding and sensitive to scanner‐specific gradient imperfections and k‐space errors^15^, ^16^ that can give rise to complex image artifacts and signal nonuniformity that can confound quantitative applications.^15^, ^17^

As an alternative, we propose a 3D Cartesian gradient‐echo acquisition approach that combines two separate half‐k‐space readouts to enable UTE imaging with full symmetric Cartesian k‐space sampling. Double half‐echo methods have been reported for fluorinated and hyperpolarized gas studies,^18^, ^19^, ^20^ termed the X‐centric approach, for sodium imaging^21^ and also for proton imaging,^22^ but limited to either 2D imaging and without UTEs. The primary goal of the current study was to develop and illustrate a robust 3D double half‐echo Cartesian UTE method for ^1^H spin‐density weighted imaging of the lung parenchyma, with both breath‐hold and respiratory navigator free‐breathing options for patient‐friendly imaging. The Cartesian double half‐echo approach was compared to the existing yarnball UTE method^13^, ^23^ in healthy subjects, which has previously been validated for quantitative LWD imaging. Free‐breathing and breath‐hold double half‐echo UTE images were compared at 1.5T and 2.89T in healthy participants along with illustrative clinical cases including comparison to CT scans.

## 2 METHODS

### 2.1 Pulse sequence design

**FIGURE 1:**
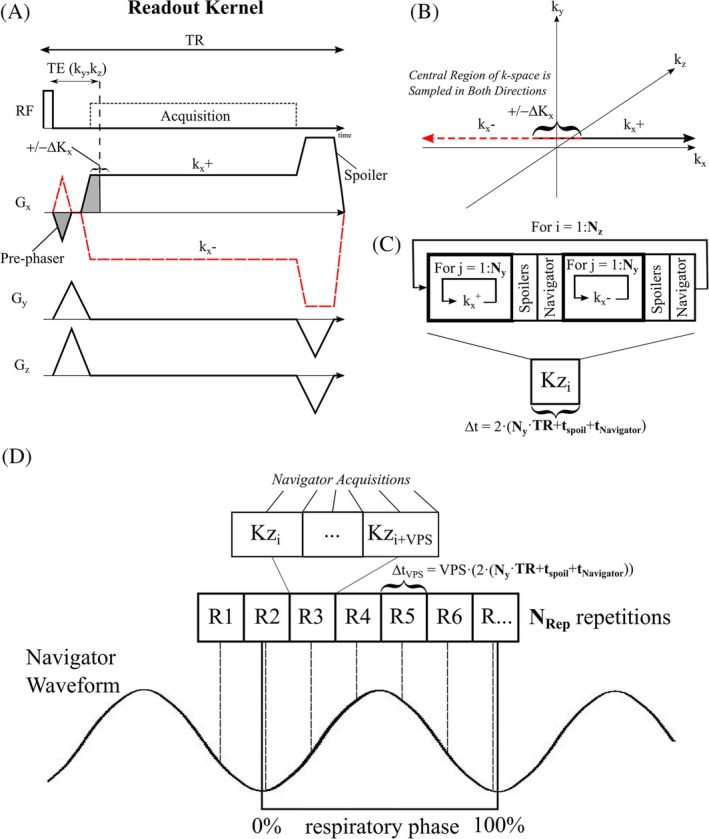
**Double half‐echo pulse sequence:** (A) Readout kernel showing positive readout (k_x_^+^) and negative readouts (k_x_^−^) and conventional k_y_ and k_z_ phase encoding. (B) A small read encoding pre‐phaser shown in A) ensures overlap of the central portion of k‐space over a region of ±ΔK_x_. (C) k‐space acquisition order for all breath‐hold double half‐echo acquisitions. The navigator is a center of k‐space acquisition which is only included for free‐breathing studies. (D) k‐space acquisition order for all free‐breathing double half‐echo acquisitions. A segment of k‐space (VPS, views per segment; k_z_ lines) is repeated N_Rep_ times to provide several samples over each respiratory cycle.

Figure 1A displays the proposed implementation of the 3D double half‐echo gradient‐echo pulse sequence. Positive readouts covering half of k‐space, k_x_^+^, and matched negative readouts, k_x_^−^, were acquired separately for each k_y_ and k_z_ phase‐encoding step (Figure 1A). A small readout gradient pre‐phaser enabled 50% + ΔK_x_ coverage for both readout directions for redundant sampling of the middle of k‐space over ±ΔK_x_ (Figure 1A,B). This redundant sampling of the central portion of k‐space enabled subsequent offset correction due to gradient delays by shifting k_x_^−^ relative k_x_^+^ (Figure 1B) without inducing gaps in the middle of k‐space. The minimum TE (time from the end of radiofrequency pulse to k_x_ = 0) was used for each k‐space location, TE(k_y_, k_z_), with the shortest times, TE_Min_, for k_y_ = 0 and k_z_ = 0. The TR was held constant at the longest value corresponding to the maximum k_y_ and k_z_ values.

Figure 1C illustrates the k‐space ordering for k_x_ and k_y_ space (N_y_ total points in the y‐direction) for a single k_z_ point, referred to collectively as Kz_i_. Within each Kz_i_ unit, the k_x_^+^ readouts were repeated for all N_y_ phase‐encoding steps (for j = 1:N_y_) followed by the k_x_^−^ readouts for the same N_y_ phase‐encoding steps, all for a single k_z_. Additional spoiler gradients (t_spoil_) between the separate sets of k_x_^+^ and k_x_^−^ acquisitions (i.e. every N_y_ acquisitions) minimized the formation of stimulated‐echoes from the reversal of the readout gradient polarity in transitioning from k_x_^+^ to k_x_^−^ acquisitions (Figure 1C). The Kz_i_ unit, with a duration of 2·(N_y_·TR + t_spoil_ + t_Navigator_) was repeated N_z_ times to sample k_z_ space, for a total acquisition time of 2·N_z_·(N_y_·TR + t_spoil_ + t_Navigator_), without image acceleration. The time for a center of k‐space navigator, t_Navigator_, is only included for free‐breathing acquisitions as detailed below.

Prior to combination of the forward and backward half‐echoes to fill k_x_‐space a variable k‐space offset (correction) between the two acquisitions was employed to correct for relative delays from gradient imperfections.^24^, ^25^, ^26^ The offset was determined in a short calibration pre‐scan using the phase‐difference between forward and backwards readouts^27^ as is routinely performed for echo‐alignment with EPI. The pre‐scan, acquired over 40 ms just prior to image acquisition, was built into the pulse sequence and the calculated k‐space correction (ΔK_Cal_.) was applied to every negative (k_x_^−^) acquisition in real‐time during data collection. The overlapping data points from the forward and backward acquisitions in the ΔK_x_ region (Figure 1B) were averaged following offset correction.

*Respirator Navigator for Free‐Breathing Acquisitions –* Similar to methods previously detailed for free‐breathing non‐Cartesian imaging approaches, a center of k‐space navigator acquisition was used to retrospectively assign a respiratory phase to all k‐space data prior to reconstruction (acquired every N_y_ lines, Figure 1C).^13^, ^28^ Segments of k‐space were repeated over complete respiratory cycles ensured full k‐space coverage at any targeted respiratory phase (Figure 1D). Each segment, defined as R1, R2, … in Figure 1D comprised multiple k_z_ lines (VPS lines, views per segment) yielding a sampling interval of Δt_VPS_ = VPS·(2·[N_y_·TR + t_spoil_ + 3·TR]) and a total duration of™_Rep_ = N_Rep_·VPS·(N_y_·TR + t_spoil_ + 3·TR), which is selected to be longer than the duration of expected breathing cycle (Figure 1D). The total acquisition time is N_z_/VPS™_Rep_, which is equal to the number of segments, N_z_/VPS, multiplied by the duration of the repeated segments, T_Rep_.

For free‐breathing studies, the center of k‐space navigator waveform was used to identify windows of k‐space to include in the final image reconstruction.^13^, ^28^ As previously detailed, the respiratory waveform for each receiver coil was filtered to remove noise and principal component analysis was used for optimal coil combination.^13^ Reconstructions targeted the functional residual capacity (FRC) respiratory phase, which is the minimum lung volume over the respiratory cycle. Repeated segments of k‐space accepted in the final image were averaged prior to reconstruction. Calculation of the navigator waveform and identification of the accepted k‐space data was performed immediately following image acquisition. On scanner reconstruction used the Siemens advanced reconstruction prototyping work‐in‐progress (FIRE, WIP 070) to run custom python software.

### 2.2 Image acquisition: parameters

Imaging data were acquired on Siemens Prisma 2.89T and Aera 1.5T scanners (Erlangen, Germany) using spine and body arrays (36 total coils) for signal reception. Coronal orientations were used for all acquisitions with the readout in the head‐to‐foot direction, phase‐encoding right‐to‐left and slice‐encoding from chest‐to‐back with a FOV of 400 mm (readout) by 500 mm (phase‐encoding) by ≥280 mm (slice‐encoding). ΔK_x_ was set to one k_x_ point for the prescribed field of view (i.e., two k_x_‐points for the native oversampled readout) for a total of 5 overlapping data points around the middle of k‐space for the k_x_^+^ and k_x_^−^ readouts (Figure 1A,B). All scans used 64 readout points for positive and negative readouts (128 total), 160 phase‐encoding and typically 56 slice‐encoding steps, for an acquired resolution of 3.125 mm by 3.125 mm by 5.0 mm, interpolated to 2.0 mm isotropic with zero filling. A higher resolution variation included 128 readout points and 320 phase‐encoding and 112 slice‐encoding steps. A GRAPPA acceleration factor of 2 was used in the phase‐encoding direction for all acquisitions. A flip angle of 2 degrees (20us rectangular RF pulse) was used to ensure minimal T_1_‐weighting.^13^ With a readout bandwidth of 1595 Hz/pixel, TE = 140 μs at the center of k_x_‐space with an additional delay to 270 μs at the corner of k_yz_‐space (k_y(max.)_, k_z(max.)_), corresponding the maximum phase‐encoding and slice‐encoding gradient areas. The total readout duration is 640 μs (10 μs dwell time, 5 μs for the native oversampled acquired data) and TR = 1.2 ms.

RF spoiling incorporated a 50° increment and the readout gradient spoiler had an area of 60% of readout gradient (Figure 1A). An additional spoiler gradient at the transition between k_x_^+^ and k_x_^−^ readouts had a duration of t_spoil_ = 2.3 ms with a gradient amplitude of 20 mT/m (Figure 1C). The total acquisition time was 12.9 s for breath‐hold scans. All acquisition parameters were identical on 1.5T and 2.89T scanners.

Free‐breathing studies used the same parameters outlined above but with N_Rep_ = 8 and VPS = 4 (Figure 1D) for Δt_VPS_ = 4·(2·[80·1.2e^−3^ + 2.3e^−3^ + 3·1.2e^−3^]) = 0.84 s resolution over the breathing cycle for a total duration of™_Rep_ = 8·Δt_VPS_ = 6.76 s and an acquisition time of 94 s. The higher resolution pulse sequence variant had a total acquisition time of 256 s.

Additionally, lung images were acquired at 2.89T using a free‐breathing non‐Cartesian yarnball UTE pulse sequence, previously validated for quantitative lung water density (LWD) imaging.^13^, ^23^ Pulse sequence parameters: 350 mm by 350 mm by 350 mm FOV, 3.5 mm by 3.5 mm by 3.5 mm resolution (interpolated to 2 mm isotropic with zero filling), TE/TR = 100 μs/2.70 ms, readout duration of 1300 μs with a 2 degree flip angle, 2738 projections with 10 repetitions for a total of 74 s acquisition during normal tidal breathing.

### 2.3 Image processing

**FIGURE 2:**
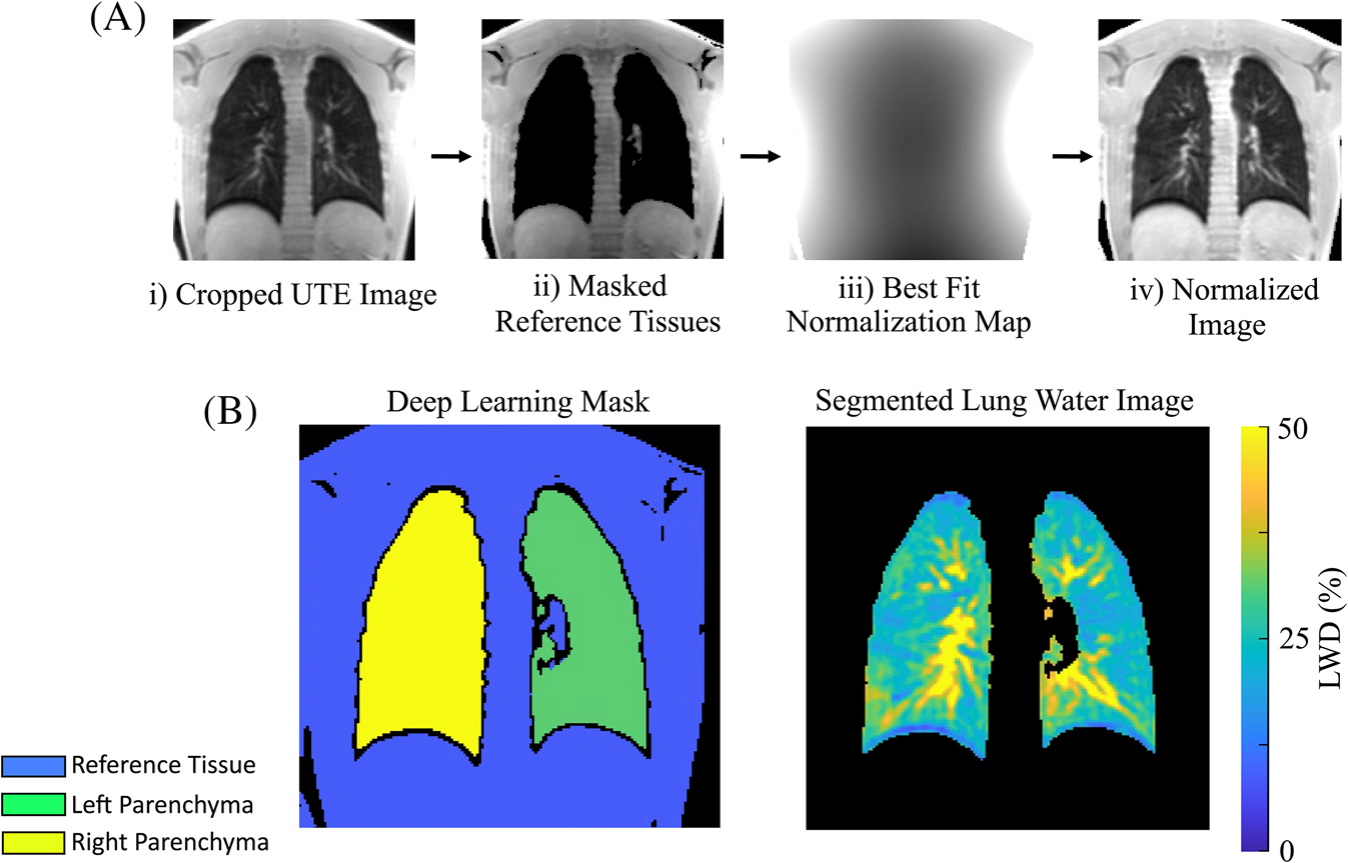
**Image processing pipeline for the generation of lung water density images** ^13^: (Ai) A cropped UTE image, (Aii) deep learning identification of reference tissue, (Aiii) calculation of the normalization map using only signals from reference tissues, and Aiv) division of input cropped UTE image by the normalization map to yield the LWD map. (B) The deep learning segmentation mask and the corresponding LWD map in the lung parenchyma. 1 of 94 coronal slices covering the full lung parenchyma is shown. LWD, lung water density.

All double half‐echo images were reconstructed on‐scanner and saved in dicom format. For post‐processing, all images (double half‐echo and yarnball) were normalized to eliminate surface coil shading, to yield relative LWD images (i.e., signal intensities are relative to the surrounding solid tissues as a percentage) using a previously described and validated approach (Figure 2).^13^ Briefly, acquired signal intensities in tissues surrounding the lungs (reference tissues) were fit with a low spatial frequency normalization map that was interpolated over the full field of view using least square linear regression with Tikhonov regularization. Division by the normalization map yielded signal intensities in normalized units, as a percentage of the reference tissues. Reference tissue and lung tissue were identified with a user‐independent machine learning segmentation approach.^29^ The nnU‐Net model is available for download at https://zenodo.org/records/15215411. Illustrative images over processing pipeline steps are shown in Figure 2.

Apparent SNR was calculated in all participants as the mean signal in user‐selected regions of interest (ROI) within the lung parenchyma divided by the SD of signal from a noise‐only region outside of the body, measured prior to image normalization. Parenchyma ROIs were traced on the coronal slice with the lowest signal intensity, approximately equidistant from the chest and back coils. SNR values for the yarnball UTE acquisitions were corrected to account for the higher spatial resolution of these acquisitions as compared to Cartesian scans.^30^ Test–retest repeatability studies were completed for all participants with repositioning on the scanner bed. A series of six free‐breathing Cartesian UTE images acquisitions were repeated in one participant, with increasing TE values of 140 μs, 300 μs, 450 μs, 600 μs, 750 μs, and 900 μs and a constant TR of 1.9 ms for all cases in order to evaluate the effect of TE on normalization procedure.

### 2.4 In‐vivo experiments

Studies were approved by the University of Alberta Health Research Ethics Board and written informed consent was obtained from all study participants. Lung images were acquired in 10 healthy volunteers (10 male, ages 18–44 years) including: (i) free‐breathing double half echo at 2.89T and (ii) 1.5T, (iii) end‐expiration breath‐hold double half‐echo at 2.89T and (iv) free‐breathing yarnball studies at 2.89T. Images from all free‐breathing acquisitions were reconstructed at functional residual capacity (minimum lung volume during restful tidal breathing).

Additionally, free‐breathing double half‐echo acquisitions were performed in two patients with sarcoidosis at 1.5T with matched CT scans, which were also acquired during free‐breathing. Finally, free‐breathing double half‐echo acquisitions were used to compare LWD in a healthy participant and a patient with heart failure at 1.5T.

### 2.5 CT acquisitions

Non‐contrast CT scans were performed on two patients with sarcoidosis during tidal breathing (Discovery MI PET/CT system, GE HealthCare). CT images were converted from Hounsfield units (HU) to normalized units according to (HU + 1000)/10 to yield zero intensity for air (−1000 HU corresponds to 0 normalized units) and approximately 100 units for solid tissues (0 HU corresponds to 100 normalized units).

### 2.6 Numerical simulations (point spread function)

The effects of $T_{2}^{*}$ decay on the point spread function (PSF) for the lung parenchyma was calculated. A $T_{2}^{*}$ of 800 μs^2^, ^3^ was used for all simulations with times after excitation ranging from 140 μs at the true center of k‐space (TE) with an additional 130 μs to the edge of k_y_, k_z_ space, and an additional 640 μs to the edge of k_x_, in the readout direction. The PSF included the effects of a Kaiser window filter with Beta = 2 used for all in‐vivo reconstructions.

### 2.7 Statistical analysis

LWD, lung volume, and SNRs for the four different acquisition approaches were compared using analysis of variance (ANOVA), with Bonferroni correction to control for multiple comparisons and Shapiro–Wilk test to confirm normality of the residuals. Reproducibility of LWD, using the test–retest acquisitions, were assessed using the intraclass correlation coefficient (ICC).

## 3 RESULTS

### 3.1 In‐vivo

**FIGURE 3:**
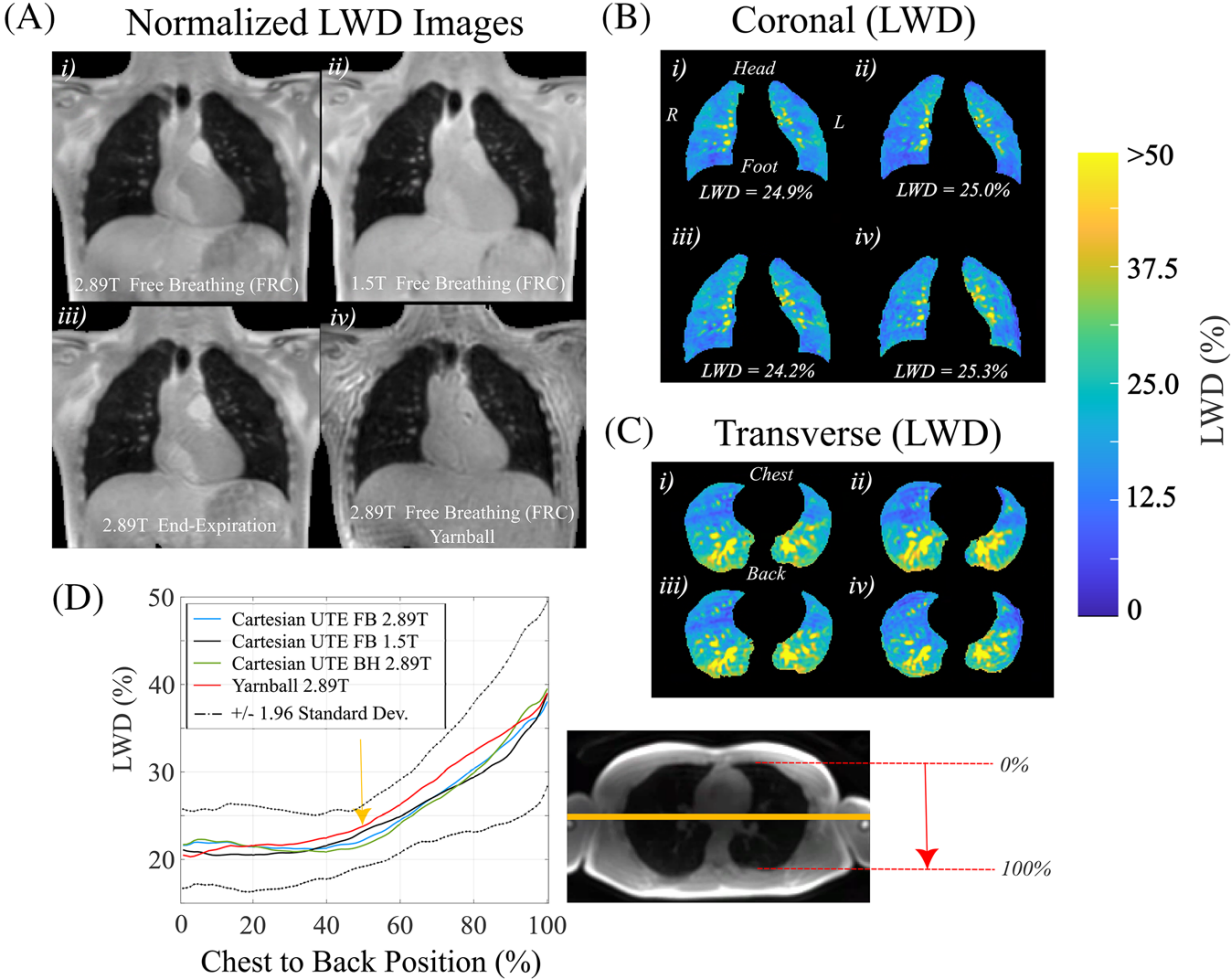
**Illustrative double half‐echo LWD images for the four different acquisition approaches**. (A) Coronal slices in one participant for free‐breathing (2.89T, 1.5T) and breath‐hold (2.89T) Cartesian double half echo acquisitions and the free‐breathing yarnball (2.89T) acquisition (FRC – functional residual capacity, minimal lung volume during tidal breathing). (B) Coronal LWD images in A) are shown with segmentation of lung parenchyma and a narrowed window level, with corresponding transverse LWD images in (C). (D) Regional LWD values for chest to back locations for all four pulse sequences from (A)–(C) including the 95% confidence intervals for all four sequences. LWD values at each spatial location correspond to the average value of all pixels in the corresponding coronal slice at that location, indicated by the yellow bar for one sample location at the 50% spatial location. LWD, lung water density.

Illustrative LWD images from one volunteer for all four pulse sequence variants shows the similar image appearance and average LWD for all cases (Figure 3). LWD images for a single coronal slice (Figure 3A) and corresponding segmented lung parenchyma (Figure 3B) as well as transverse slices of lung parenchyma (Figure 3C) highlight the similar spatial patterns of LWD and machine learning segmentation masks for the four different acquisitions. The pattern of elevated LWD toward the back was similar for all four pulse sequences (average values and 95% confidence interval for all 10 participants, Figure 3D). Reported LWD values as a function of chest to back location in Figure 3D included the average LWD value within the coronal slice at each slice location (orange line in Figure 3D), with positions normalized from 0% to 100% of the chest‐to‐back dimension of the lung parenchyma. Average whole lung LWD values, lung volumes, SNRs and ICC values for test–retest reproducibility are shown in Table 1.

**TABLE 1:** Average lung parameters for all pulse sequences (*N* = 10).

	DHE 2.89T	DHE 1.5T	DHE 2.89T	YB 2.89T
Breathing Maneuver	FB (FRC)	FB (FRC)	BH (EE)	FB (FRC)
LWD (%)	27.1 ± 2.5	26.7 ± 2.9	27.1 ± 2.1	27.7 ± 2.7
Lung volume (mL)	2640 ± 657	2688 ± 586	2612 ± 541	2605 ± 593
SNR (ratio)*	21.8 ± 1.7	18.4 ± 1.4	15.1 ± 1.0	20.7 ± 1.1
Test–retest, ICC	0.98	0.94	0.97	0.97

Abbreviations: BH, breath‐hold; DHE, double half‐echo Cartesian; EE, end‐expiration; FB, free‐breathing; FRC, functional residual capacity; ICC, intraclass correlation coefficient; LWD, lung water density; YB, yarnball.

* *p* < 0.05 for all pulse sequence comparisons except DHE FB at 2.89T versus YB at 2.89T.

**FIGURE 4:**
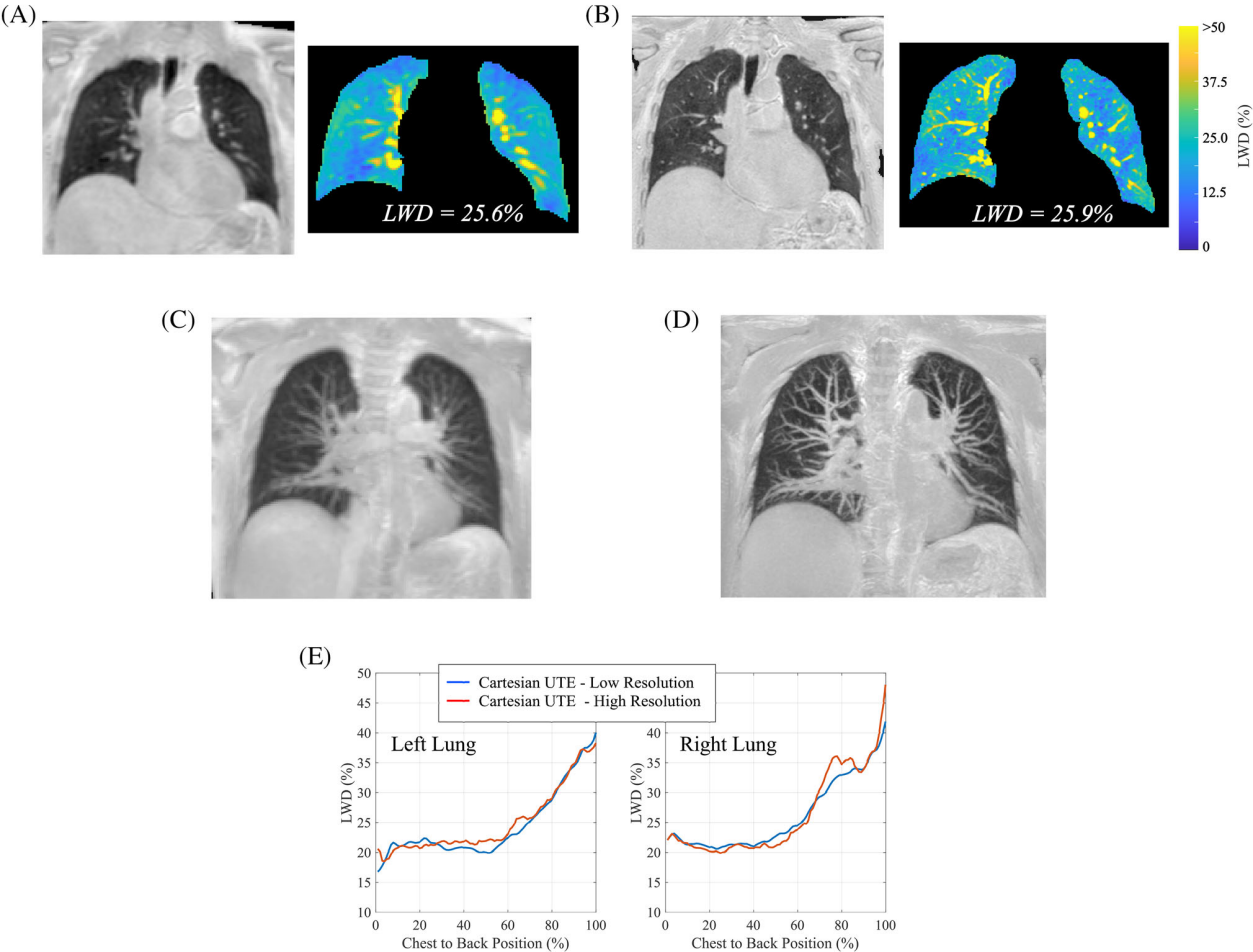
**Cartesian double half echo images with increased spatial resolution**. Comparison of free‐breathing Cartesian double half‐echo LWD images in one participant with (A) the default spatial resolution and (B) double spatial resolution in all three dimensions. Also included are the segmented lung parenchyma for the same coronal slice with a narrowed window level. MIPS (20 mm projection thickness) for the corresponding images are shown in (C) and (D). (E) Spatial variation in LWD from chest to back locations are plotted separately for the left and right lungs, for both default and higher spatial resolutions acquisitions. (See Figure 3D for a description of the chest to back signal derivation.) LWD, lung water density.

Figure 4 compares conventional (Figure 4A) and higher resolution (Figure 4B) double half‐echo free‐breathing images for one participant. All other images in the current study were acquired at the conventional resolution. One illustrative coronal slice with normalized LWD values is shown for each case (left), including the application of the segmentation mask and a reduced range of signal intensity values to highlight the similarity of LWD for the two different resolutions (right). A maximum intensity projection (MIP) for these two cases in Figure 4C,D, over a thickness of 20 mm in the chest to back direction, illustrates the improved blood vessel visibility with higher spatial resolution. Average lung water density as a function of position from chest to back, for both the left and right lungs (Figure 4E), illustrates the similar LWD values between lower and higher resolution acquisitions (See Figure 3D for a description of the chest to back signal derivation).

**FIGURE 5:**
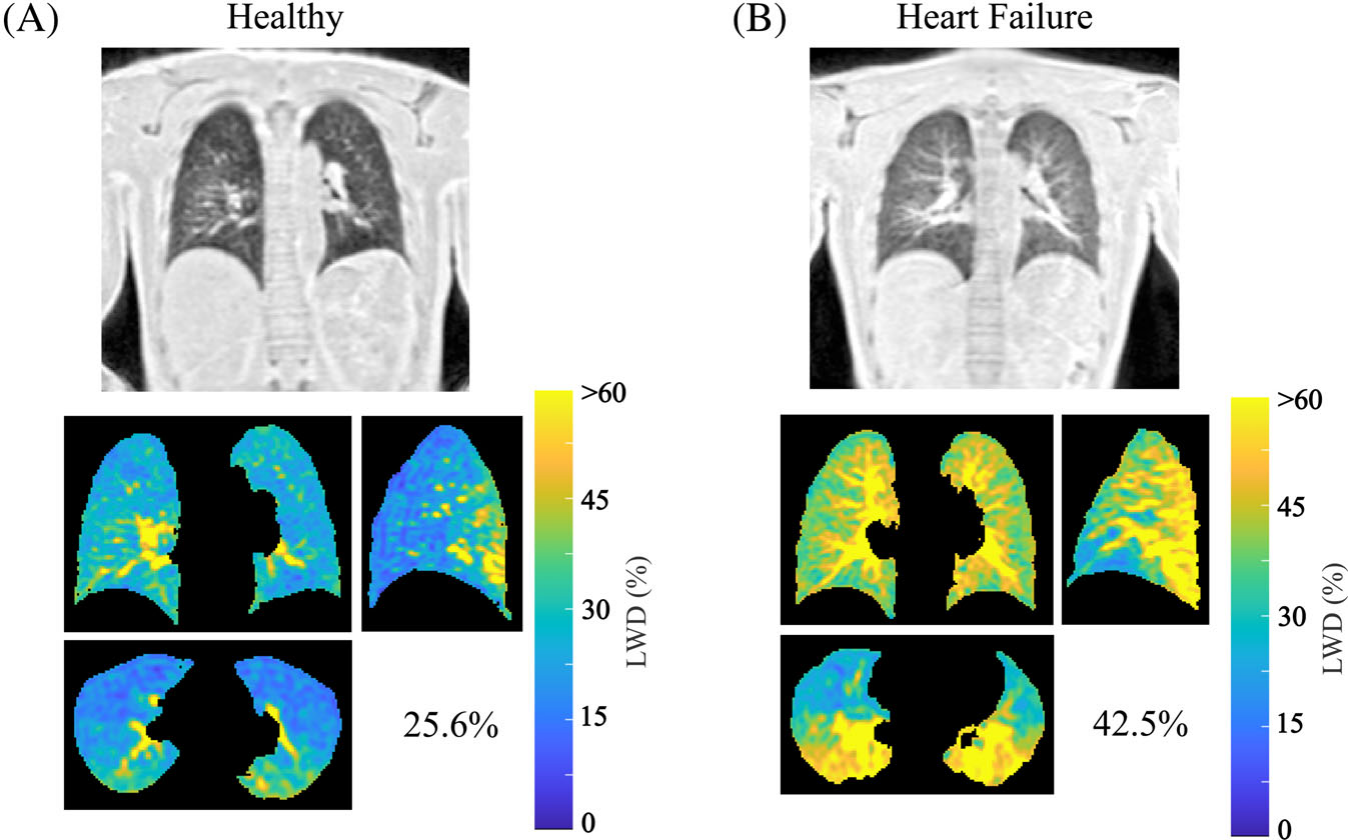
**Increased LWD in a patient with heart failure**. Double half‐echo LWD images (coronal, sagittal and axial views) in (A) a healthy participant and (B) a patient with heart failure highlight the increased parenchyma water content in the heart failure patient, 42.5% on average as compared to 25.6% in the healthy participant. MRI studies were performed at 1.5T.

Figure 5A,B compare free‐breathing double half‐echo MR images (1.5T) in a healthy participant and a patient with heart failure in representative coronal, sagittal, and transverse slices. The patient has elevated whole lung average LWD values of 42.5% as compared to 25.6% in the healthy participant.

**FIGURE 6:**
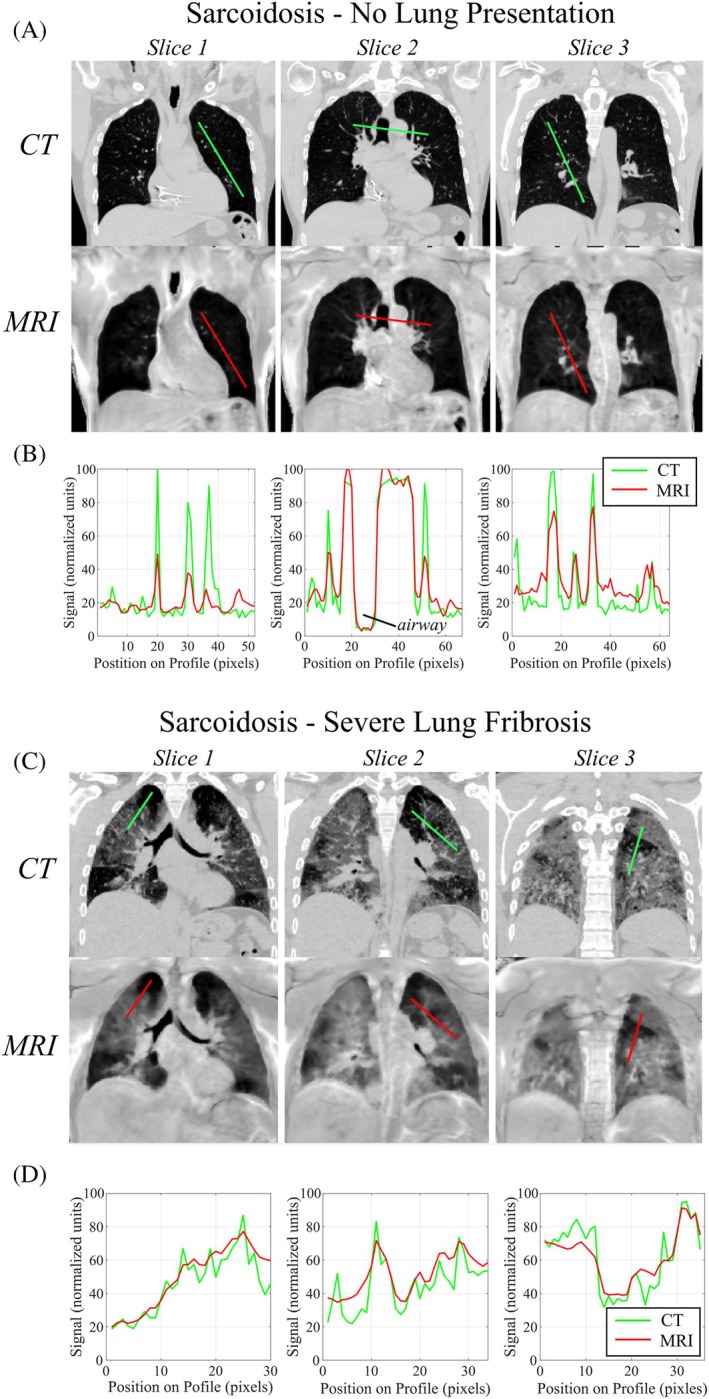
**LWD imaging in two patients with sarcoidosis – comparison with CT**. Comparison of CT and double half‐echo images (three sample coronal slices) in two cases of sarcoidosis, with (A) no lung presentation and (C) severe lung fibrosis. Signal intensity profiles in (B) and (D) along the green (CT) and red (MRI) lines illustrate the similar normalized signal intensities between the two modalities.

Comparison of matched CT and normalized free‐breathing double half‐echo MR images (1.5T) in two patients with sarcoidosis illustrate the close agreement between MRI and CT in cases with no imaging findings in the lungs (Figure 6A) and severe fibrosis (Figure 6C). Signal intensity profiles from three representative coronal slices from each patient (Figure 6B,D) illustrate the good agreement between normalized MRI and CT signal intensities in both the healthy and fibrotic lung parenchyma.

**FIGURE 7:**
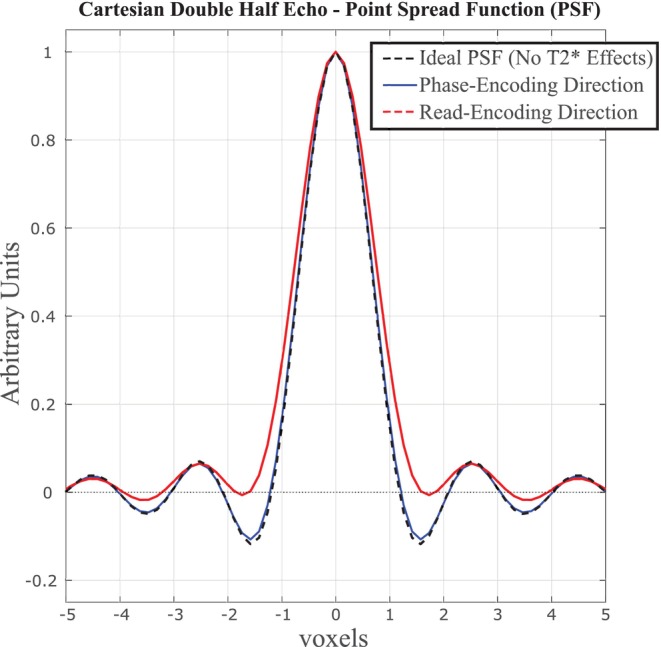
**Calculated PSF in lung parenchyma for the Cartesian double half echo pulse sequence**. Numerically calculated PSF for lung parenchyma ($T_{2}^{*}$ = 800 μs) for the Cartesian double half echo pulse sequence using in‐vivo acquisition parameters including the effects of the Kaiser window filter (Beta = 2) used for in‐vivo filtering of k‐space data.

**FIGURE 8:**
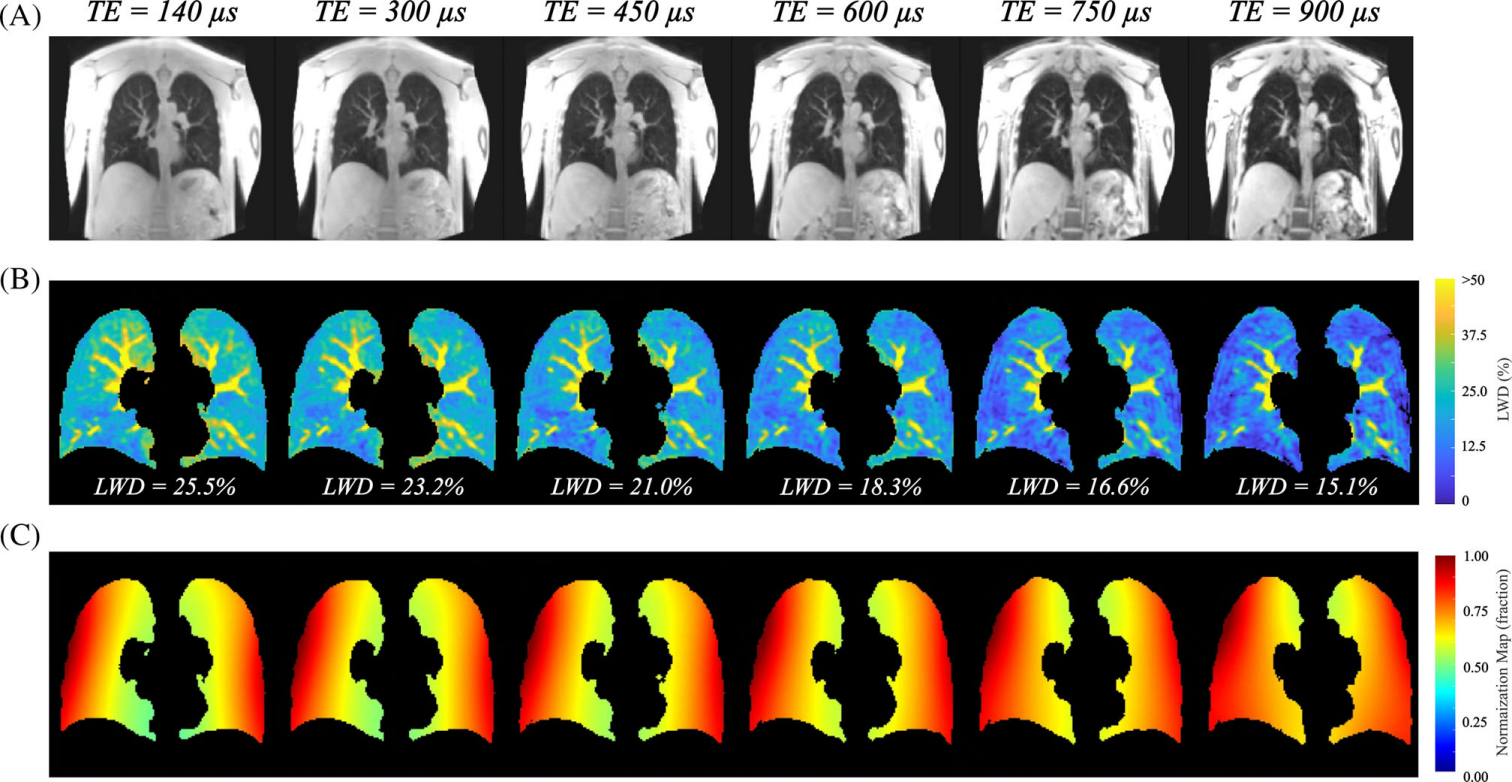
**Cartesian double half echo UTE images as a function of TE**. (A) Unprocessed UTE images show signal decay in the lungs, in the stomach region and regions of fat and water overlap (vertebrae and between muscle groups) with increasing echo time. (B) Segmented LWD images illustrate significant $T_{2}^{*}$ decay with increasing TE. (C) The normalization map used to generate LWD images is similar for all TE times. One coronal slice from 135 slices is shown. LWD, lung water density.

Calculated point spread functions in the phase‐encoding and read‐encoding directions illustrate negligible effects of lung parenchyma signal decay ($T_{2}^{*}$ = 800 μs) on the features of the point spread function, for the pulse sequence parameters used in the current study (Figure 7). Increasing the TE beyond the shorter UTE values of 140 μs, up to 900 μs, significantly reduced calculated LWD values as expected, due to $T_{2}^{*}$ decay, but with only minor effects on the calculated normalization field used to convert acquired images to LWD units (Figure 8).

## 4 DISCUSSION

We presented a 3D ultrashort echo time double half‐echo Cartesian pulse sequence for application in lung water density imaging. Both breath‐hold duration (13 s) and navigator free‐breathing pulse sequence variations (94 s) had sufficient spatial resolution and SNR for quantification of LWD at both 1.5T and 2.89T field strengths. Reported SNR values reflect the minimum values within the lungs, with increasing SNR toward the chest and back, closer to the receiver coils. The free‐breathing variation offers the advantage of no required patient compliance, increased SNR due to the effects of averaging, and the option for higher spatial resolution that would preclude breath‐hold duration acquisitions. An intermittent center of k‐space acquisition was added to enable retrospective selection of data for reconstruction of images at functional residual capacity. Comparison of LWD images for the proposed Cartesian approach and a previously validated yarnball non‐Cartesian UTE pulse sequence^13^, ^23^ illustrated similar image appearance and quantitative LWD values measured globally and regionally. Whole lung average LWD and lung volumes were shown to be independent of the acquisition approach with a consideration of breathing maneuver (breath‐hold or free‐breathing), field strength, or Cartesian or non‐Cartesian k‐space trajectory.

The yarnball gradient waveforms were compensated for the gradient finite impulse response of the MRI system using in‐house‐developed methodology.^14^ The only corrections applied to the Cartesian UTE gradient waveforms was the delay between the positive and negative readouts measured from a calibration pre‐scan, which was calculated and applied in real‐time for each scan.

Higher acceleration rates, beyond the standard rate 2 GRAPPA used in the current study could reduce scan times for the double half‐echo approach, but future studies are necessary to determine tradeoffs between acquisition time, SNR and potential artifacts in the lower SNR regions within the lungs. The required readout delay, to accommodate the phase‐encoding gradients, shifted the acquisitions by an additional 130 μs at furthest extent of k_x_ and k_y_ values, which was shown to have negligible effects on the point spread function in those directions. Similarly, the relatively short readout duration of 600 μs had a negligible effect on the point spread function in the x‐direction.

Application of the free‐breathing double half echo approach in a clinical case of heart failure illustrated the quantification of globally elevated LWD associated with cardiogenic pulmonary edema. This is in‐line with previous studies in which MRI‐derived LWD, measured with non‐Cartesian UTE methods, was shown to be elevated and to be prognostic in heart failure^5^, ^8^ as well as being elevated in patients at‐risk for heart failure,^31^ largely independent of conventional measures of heart function. Additionally, the application of the free‐breathing double half echo approach for the detection of pulmonary fibrosis was illustrated in two patients diagnosed with sarcoidosis, a disease with a 20% prevalence of pulmonary fibrosis.^32^ Qualitatively, overall features were very similar between MRI and matched CT scans for patients with and without pulmonary fibrosis, and quantitative comparison of MRI‐derived LWD and normalized CT scans illustrated the close agreement between LWD and normalized Hounsfield units (i.e., where air has a value of zero and water has a value of 100).

This study has a number of limitations. The relatively low spatial resolution was selected in part to provide short acquisition times and sufficient SNR for quantification of LWD at the expense of blurring of pulmonary vessels and thus partial volume effects for quantification of lung parenchyma water density. SNR calculations were approximate and would ideally have included noise‐only scans and other methods to account for the effects of non‐Cartesian sampling and parallel imaging.^33^ The use of a center of a k‐space navigator for free‐breathing acquisitions is inefficient and approximate and will be susceptible to blurring artifacts depending on breathing patterns. More advanced post‐processing approaches^34^, ^35^ could improve efficiency and image quality for free‐breathing acquisitions.

## 5 CONCLUSIONS

A double half‐echo Cartesian ultrashort echo time pulse sequence for quantitative imaging of the lung parenchyma was developed and evaluated at 1.5T and 2.89T field strengths. Patient‐friendly breath‐hold or short navigator gated variations of the sequence yielded similar results with sufficient signal to noise for quantification of LWD. A built‐in calibration for echo alignment enabled real‐time corrections of gradient delays and fast on‐scanner image reconstruction without the need for k‐space gridding.
